# Smoking cessation in Chinese patients undergoing thoracic surgery: A multicenter prospective observational study

**DOI:** 10.18332/tid/175639

**Published:** 2024-01-10

**Authors:** Jianxing He, Dingpei Han, Kun Qian, Weijie Guan, Ge Zhang, Weiqing Lu, Hecheng Li, Xiuyi Zhi

**Affiliations:** 1 Department of Thoracic Surgery, Guangzhou Institute of Respiratory Disease, The First Affiliated Hospital of Guangzhou Medical University, Guangzhou Medical University, Guangzhou, China; 2 State Key Laboratory of Respiratory Disease, National Clinical Research Center for Respiratory Disease, Guangzhou Institute of Respiratory Health, The First Affiliated Hospital of Guangzhou Medical University, Guangzhou Medical University, Guangzhou, China; 3 Department of Thoracic Surgery, Ruijin Hospital, Shanghai Jiao Tong University School of Medicine, Shanghai, China; 4 Department of Thoracic Surgery, Xuanwu Hospital, Capital Medical University, Beijing, China; 5 Lung Cancer Initiative, Johnson & Johnson Enterprise Innovation, Shanghai, China

**Keywords:** smoking cessation, lung surgery, post-operative complication, smoking pattern, smoking relapse

## Abstract

**INTRODUCTION:**

The multicenter CHAMPION study aimed to assess the impact of smoking cessation on post-operative complications (PCs) and smoking cessation patterns in Chinese patients undergoing lung surgery.

**METHODS:**

Patients undergoing elective lung surgery were prospectively enrolled from three major tertiary centers in China. Patients were categorized as smokers or quitters before surgery. Baseline characteristics and smoking status were analyzed. The incidence of PCs and pulmonary PCs (PPCs), smoking relapse rate, and causes within six months post-operatively were investigated. The questionnaire was conducted in all patients and 30 healthcare professionals (HCPs), regarding the awareness and effectiveness of smoking cessation methods.

**RESULTS:**

Of the 276 enrolled patients, 213 (77.2%) were smokers and 63 (22.8%) were quitters; 76.4% were diagnosed with primary lung cancer. PCs occurred in 13.8% of patients, with similar proportions in smokers (14.1%) and quitters (12.7%). PPCs occurred in 9.8% of patients with no significant differences between smokers and quitters (9.4% vs 11.1%, p=0.70). At six months, 9.2% of patients relapsed, with a lower rate in quitters compared to smokers (3.3% vs 11.0%, p=0.01). HCPs exhibited higher awareness of smoking cessation methods than patients. Perceived effectiveness of smoking cessation methods from the patients were low.

**CONCLUSIONS:**

In patients undergoing lung surgery with a low risk of PCs, active smoking does not significantly increase the risk of PCs or PPCs relative to quitters, suggesting that there is likely no need to postpone lung surgery for those who have not yet quit smoking. However, further large-scale studies are necessary to confirm these findings.

## INTRODUCTION

China has the largest number (about 316 million adults) of active smokers in the world (accounting for 30% of smokers worldwide)^1^. Despite the launch of the WHO Framework Convention on Tobacco Control in 2016, the practical implementation in China has not been ideal^2^. The prevalence of smoking in China remains high at 26.6% in 2018^3^. Most Chinese smokers (75.6%) had made no plans to quit, and about 20% of adult smokers quit smoking in 2018^4,5^. The most frequently reported reason by smokers for quitting smoking is health preservation^4^.

Cigarette smoking is the predominant risk factor for lung cancer, accounting for almost 90% of cases^6,7^. Smoking cessation may not only decrease the risk of tobacco-related lung diseases but has a survival benefit even among patients who have previously been treated^8,9^. For patients undergoing lung surgery, smoking is an important reversible risk factor for post-operative complications (PCs) and pulmonary PCs (PPCs), although the impact of smoking cessation varies based on the different surgical types and populations^10^. A recent systematic review concluded that smoking cessation should be recommended pre-operatively among patients scheduled for thoracic surgery^11^.

The evidence of current smoking cessation patterns and the impact of smoking cessation in Chinese patients undergoing lung surgery is scarce. Hence, this multicenter prospective China Observational Study of Smoking Cessation Patterns in Patients undergoing Lung Surgery (CHAMPION) aimed to investigate the differences in the incidence of PCs and PPCs in smokers and quitters, post-operative smoking relapse, and the use of nicotine replacement therapy (NRT) or non-NRT by the patients or their healthcare professionals (HCPs).

## METHODS

### Study design

This multicenter, prospective, observational study enrolled patients from three representative thoracic surgical centers in: Xuanwu Hospital, Medical University; Ruijin Hospital, Shanghai Jiao Tong University School of Medicine; and First Affiliated Hospital of Guangzhou Medical University, from May 2020 to December 2021. The study was approved by the institutional Ethics Committees/Institutional Review Boards and was performed in compliance with the Declaration of Helsinki. Written informed consent was obtained from all participants.

**Figure 1:**
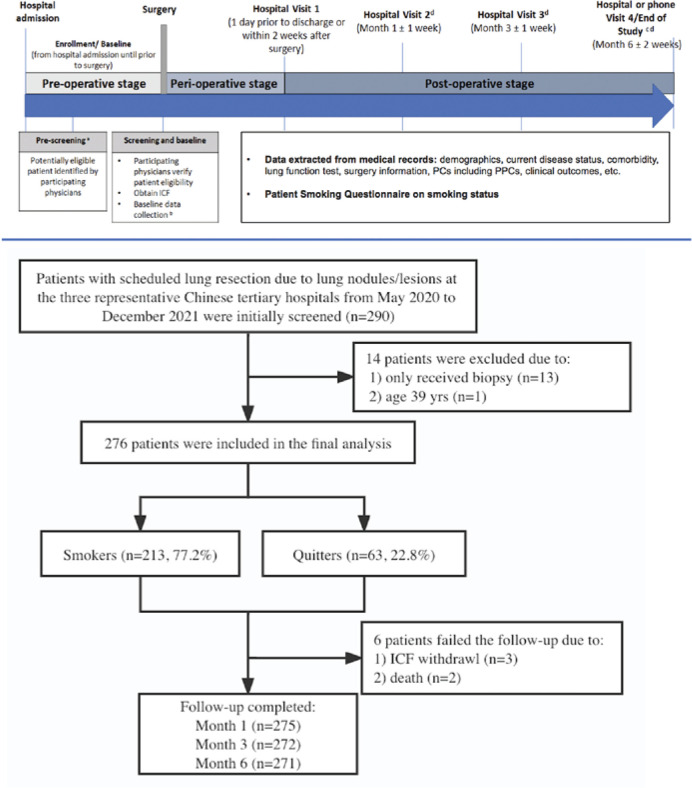
Flow diagram of the study design

Eligible patients were aged 40–80 years, scheduled for elective lung resection due to pulmonary lesions (e.g. nodule, pulmonary bullae), met the definitions of smokers or quitters, and were willing to participate in the study. Patients were excluded if they had emergency lung surgery due to an accident or injury, or were unable to complete the 6-month follow-up post-operatively. The study design is shown in Figure 1.

Smokers were defined as those who had been smoking ≥5 cigarettes per day for ≥3 years within 3 months prior to the scheduled surgery. Quitters were defined as those who had been smoking ≥5 cigarettes per day for ≥3 years and quitting smoking between 3 months and 5 years prior to the scheduled surgery. Relapse was defined as smoking re-initiation at 1 day prior to discharge or within 2 weeks after surgery.

Additionally, 30 HCPs (10 from each site) were enrolled to complete the questionnaire of smoking cessation pattern, after providing a written informed consent form.

### Data collection

Demographic and clinical characteristics data were collected from medical records. The incidence of perioperative PCs, and PPCs were assessed at 1 day prior to discharge or within 2 weeks after surgery, and at Months 1, 3, and 6 post-operatively (Supplementary file Table S1). The smoking status, smoking cessation attempts, previous uptake of smoking cessation medications, and any advice or service received from HCPs, and patient satisfaction, were collected from a patient-oriented questionnaire. All patient-level information was recorded in an electronic data capture system.

For HCP, the information on smoking cessation methods the HCPs had used, their awareness of the benefits of smoking cessation for patients, smoking cessation advice or service they had provided to patients, and the awareness of the available medicinal smoking cessation products such as nicotine replacement therapy (NRT), were collected in the HCP-oriented questionnaire, which was completed during patient enrollment. The relapse rate during the 6-month follow-up and the causes of relapse were also investigated.

### Statistical analysis

We estimated that 300 patients would be needed based on previously reported PC incidence of approximately 20% among smokers undergoing pulmonary surgeries^12^ with its 95% confidence interval (CI) 16% to 25%. Continuous variables were presented as mean with standard deviation (SD). Categorical data were expressed as frequencies and percentages. For the baseline clinical characteristics, two-sample t-test for continuous variables and Fisher’s exact test for categorical variables were used to detect the difference between two groups. For PC and PPC incidence, the risk difference and two-sided 95% CI calculated by the Wald method are presented. All analyses were performed using the JMP software package (version 11, SAS Institute Inc.). A two-tailed p<0.05 was considered statistically significant.

## RESULTS

### Demographics and baseline characteristics

We enrolled 276 patients who underwent lung resection for pulmonary nodules or lesions, with 213 (77.2%) current smokers and 63 (22.8%) quitters. Five patients were lost to follow-up and 271 (98.2%) completed the 6-month follow-up (Figure 1).

The mean age of the whole study cohort (98.9% males) was 59.4 years. The study cohort had a mean smoking history of 35.4 years (21.2 cigarettes per day). The baseline characteristics including age, gender, residence, BMI, education level, and comorbidities, were comparable between smokers and quitters (Table 1). The mean Fagerström test for nicotine dependence (FTND) score was 4.1 among smokers.

**Table 1:** Clinical characteristics by smoking cessation in Chinese patients undergoing thoracic surgery (N=276)

*Characteristics*	*Total (N=276) n (%)*	*Smokers (N=213) n (%)*	*Quitters (N=63) n (%)*	*p*
**Demographics**				
**Age** (years), mean (SD)	59.4 (8.7)	59.3 (8.4)	59.7 (9.6)	0.79
Male	273 (98.9)	211 (99.1)	62 (98.4)	0.54
**Residence**				1.00
Rural	92 (33.3)	71 (33.3)	21 (33.3)	
Urban	184 (66.7)	142 (66.7)	42 (66.7)	
**BMI (**kg/m^2^), mean (SD)	24.1 (3.2)	24.0 (3.3)	24.4 (2.8)	0.36
Education level*				0.80
Primary school or lower	37 (13.4)	27 (12.7)	10 (15.9)	
Junior middle school	91 (33.0)	73 (34.3)	18 (28.6)	
Senior high school or equivalent	88 (31.9)	68 (31.9)	20 (31.7)	
College and higher	57 (20.7)	43 (20.2)	14 (22.2)	
**Comorbidities**				
Asthma	4 (1.4)	4 (1.9)	0 (0.0)	0.58
Chronic bronchitis	6 (2.2)	5 (2.3)	1 (1.6)	1.00
Emphysema	15 (5.4)	13 (6.1)	2 (3.2)	0.53
COPD	5 (1.8)	2 (0.9)	3 (4.8)	0.08
CAD	18 (6.5)	16 (7.5)	2 (3.2)	0.38
HTN	97 (35.1)	77 (36.2)	20 (31.7)	0.55
DM	33 (12.0)	26 (12.2)	7 (11.1)	1.00
Hyperlipidemia	10 (3.6)	6 (2.8)	4 (6.3)	0.24
Lung function*				
FEV1 (mL), mean (SD)	2677 (659.7)	2685 (650.0)	2651 (698.5)	0.74
FEV1%, mean (SD)	73.8 (11.0)	73.9 (11.0)	73.7 (11.0)	0.93
DLCO (mmol/min/kPa), mean (SD)	7.602 (3.0011)	7.596 (3.2007)	7.622 (2.2325)	0.95
PEF (L/s), mean (SD)	7.206 (2.0126)	7.190 (1.9871)	7.261 (2.1184)	0.82
**Smoking status**				
Average daily smoking number of cigarettes, mean (SD)	21.2 (10.9)	21.4 (11.3)	20.5 (9.6)	0.59
Smoking history (years), mean (SD)	35.4 (11.2)	36.0 (10.8)	33.3 (12.3)	0.10
FTND score, mean (SD)	4.1 (2.4)	4.1 (2.4)	NA	NA
**Surgical procedures**				
**VATS**	268 (97.1)	208 (97.7)	60 (95.2)	0.39
**Types of surgery**				0.08
Lobectomy	162 (58.7)	131 (61.5)	31 (49.2)	
Wedge resection	57 (20.7)	38 (17.8)	19 (30.2)	
Segmentectomy	36 (13.0)	30 (14.1)	6 (9.5)	
Other	21 (7.6)	14 (6.6)	7 (11.1)	
**Pathological diagnosis**				0.75
Primary lung cancer	211 (76.4)	160 (75.1)	51 (81.0)	
Metastatic tumor	5 (1.8)	4 (1.9)	1 (1.6)	
Other	60 (21.7)	49 (23.0)	11 (17.5)	

* With missing data. The percentages are based on the number of all patients. Two-sample t-test for continuous variables and Fisher’s exact test for categorical variables were used to detect the difference in clinical characteristics between two groups. Two-sided p-values are reported. BMI: body mass index. COPD: chronic obstructive pulmonary disease. CAD: coronary artery disease. HTN: hypertension. DM: diabetes mellitus. DLCO: diffusing capacity of lungs for carbon monoxide. FEV: forced expiratory volume. PEF: peak expiratory flow. FTND: Fagerström test for nicotine dependence. NA: not applicable. VATS: video-assisted thoracoscopic surgery.

Data of lung function test were available in 234 patients. The forced expiratory volume in one second (FEV1), FEV1% predicted, and peak expiratory flow and diffusing capacity, were balanced between smokers and quitters.

### Post-operative complications

Most patients (97.1%) underwent video-assisted thoracoscopic surgery, with lobectomy being performed in 61.5% of smokers and 49.2% of quitters, respectively; 76.4% of patients were diagnosed as having primary lung cancer and 1.8% metastatic lung tumors (Table 1).

**Figure 2:**
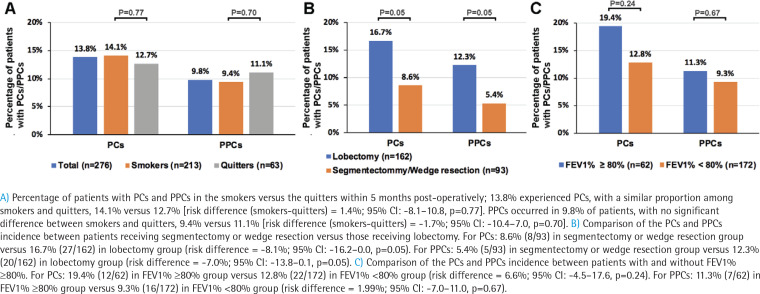
Pulmonary post-operative complications and post-operative complications by smoking status

Within 6 months post-operatively, 13.8% experienced PCs, with a similar proportion among smokers and quitters, 14.1% versus 12.7% (risk difference (smokers-quitters) = 1.4%; 95% CI: -8.1–10.8, p=0.77). The major PCs (n=10; 3.6%) included pleural effusion, pneumothorax, post procedural hemorrhage, and cerebral infarction. PPCs occurred in 9.8% of patients, with no significant difference between smokers and quitters, 9.4% versus 11.1% (risk difference (smokers-quitters) = -1.7%; 95% CI: -10.4–7.0, p=0.70) (Figure 2A). Major PPCs (n=6; 2.2%) included pleural effusion, pneumothorax, post procedural hemorrhage, and atelectasis. A list of PCs and PPCs is shown in Supplementary file Table S2. Additionally, there were no significant differences in the occurrence of PCs and PPCS between smokers and quitters at Month 1, 3 and 6, as detailed in the Supplementary file Table S3.

We next compared PCs and PPCs by stratifying patients, according to the mode of surgery. Patients receiving segmentectomy or wedge resection had a marginal significantly decreased incidence of PCs and PPCs compared with those receiving lobectomy (Figure 2B and Supplementary file Table S4). For PCs, there were 8.6% (8/93) in segmentectomy or wedge resection group versus 16.7% (27/162) in lobectomy group (risk difference = -8.1%; 95% CI: -16.2–0.0, p=0.05). For PPCs, there were 5.4% (5/93) in segmentectomy or wedge resection group versus 12.3% (20/162) in lobectomy group (risk difference = -7.0%; 95% CI: -13.8–0.1, p=0.05). Furthermore, the incidence of PCs and PPCs was not significantly different between patients with FEV1% ≥80% and those without (Figure 2C, Supplementary file Table S5). For PCs, there were 19.4% (12/62) in FEV1% ≥80% group versus 12.8% (22/172) in FEV1% < 80% group (risk difference = 6.6%; 95% CI: -4.5–17.6, p=0.24). For PPCs, there were 11.3% (7/62) in FEV1% ≥80% group versus 9.3% (16/172) in FEV1% <80% group ( risk difference = 1.99%; 95% CI: -7.0–11.0, p =0.67).

### Smoking relapses and triggers

**Figure 3:**
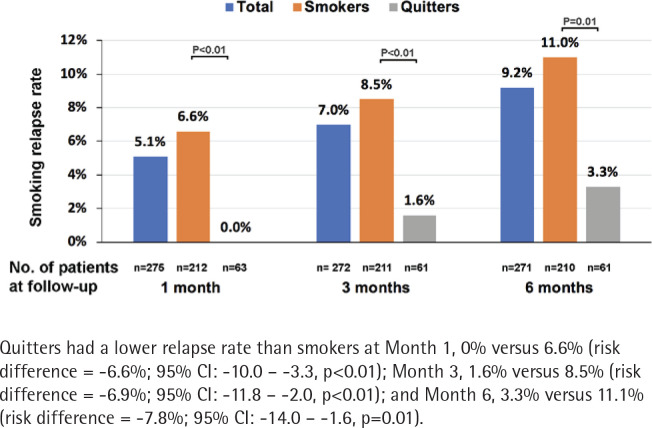
Smoking relapse rate during the 6-month follow-up

**Figure 4:**
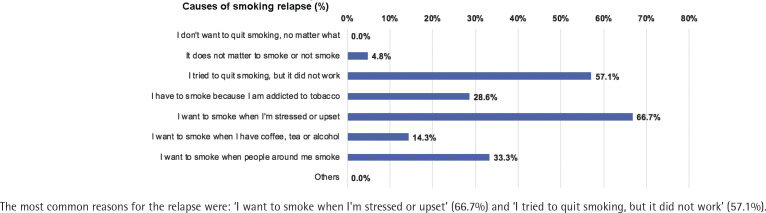
Causes of smoking relapse

The number of patients who completed follow-up at Month 1, 3 and 6 was 275, 272 and 271, respectively; 5.1%, 7.0% and 9.2% had resumed smoking at Month 1, 3, and 6, respectively. Quitters had a lower relapse rate than smokers at Month 1, 0% vs 6.6% [risk difference (quitters-smokers) = -6.6%; 95% CI: -10.0 – -3.3, p<0.01], Month 3, 1.6% vs 8.5% (risk difference = -6.9%; 95% CI: -11.8% – -2.0, p<0.01), and Month 6, 3.3% vs 11.1% (risk difference = -7.8%; 95% CI: -14.0 – -1.6, p=0.01) (Figure 3). The most common reasons for the relapse were: ‘I want to smoke when I'm stressed or upset’ (66.7%), and ‘I tried to quit smoking, but it did not work’ (57.1%) (Figure 4).

### Smoking cessation pattern

Next, we evaluated the awareness and perceived effectiveness of NRT and non-NRT smoking cessation methods by patients and HCPs. HCPs generally had a higher awareness of most smoking cessation methods than the patients. The acquaintance of non-NRT of HCPs varied from 3.3–66.7%, with more than half of HCPs having heard of or attempted to initiate the interventions such as counselling, smoking cessation materials, cessation hotline, behavior support, medication, or exercising. Nicotine patches and nicotine gum were the two major NRTs that were applied by 23.3–30% of HCPs. The awareness of smoking cessation methods, regardless of the non-NRT or NRT, was much lower in patients than in HCPs. Only 13.4% of patients attempted to use e-cigarettes, and 19.6% received behavioral support. The percentage of other methods by the patients was consistently <2.2%. Specifically, NRT of nicotine patches yielded the highest awareness (8.7%) among the patients but only 0.7% of patients had ever used nicotine patches. E-cigarette (55.4%) was the most commonly heard non-NRT among patients. Perceived effectiveness from the patients was generally low. Perceived effectiveness of NRT from HCPs was higher than that from patients (Supplementary file Figure S1).

## DISCUSSION

Despite the extensive evidence about the benefits of cigarette cessation among smokers in the general population, little is known regarding the benefits of cigarette smoking cessation among active smokers who are scheduled for lung surgery. The prospective CHAMPION study consisted of 276 patients who had an average smoking history of 35 years with a daily cigarette consumption of more than one pack; 77.2% of patients quit smoking within three months prior to the lung surgery and nearly 10% had resumed smoking post-operatively. There was no remarkable difference in the total incidence of PCs and PPCs between active smokers and quitters. However, the awareness of smoking cessation methods among patients was generally low.

PCs and PPCs are common and major causes of perioperative morbidity and mortality^13^. Major PPCs include respiratory infection, respiratory failure, pleural effusion, atelectasis, pneumothorax, bronchospasm, and aspiration pneumonitis^14^. Multiple factors affect the incidence of PPCs following thoracic surgery, and the incidence of PPCs varied from 13% to 38.8% among the different definitions and study populations^15-19^. The overall PPCs incidence in the CHAMPION study was only 9.8%, lower than previously reported. Some clinical factors could have contributed to these notable differences. First, nearly all patients in our study had undergone VATS, and VATS instead of open thoracic surgery. VATS has been shown to result in decreased rates of post-operative respiratory complications such as atelectasis, pneumonia, and respiratory failure when compared to open thoracotomy. This may be due to the smaller incision and reduced trauma to the chest wall, resulting in improved respiratory function^20^. Furthermore, VATS has been associated with lower intraoperative blood loss and reduced need for blood transfusion compared to open thoracotomy, which may contribute to a lower risk of related post-operative complications^20^. Second, sublobar resections were performed in approximately one-fifth of the patients in our study. Sublobar resections would be particularly indicated among elderly patients and in those with a high comorbidity index or reduced respiratory functional reserve. The less invasive nature of sublobar resection helps minimize the impact on lung function and overall physiological reserve, leading to lower rates of post-operative complications than lobectomy^21^. However, while sublobar resection may reduce post-operative complications, it is typically reserved for early-stage lung cancer cases with smaller tumor sizes and peripheral locations, as it may not provide the same oncological benefits as complete lobe removal^21^. Furthermore, the prevalence of coexisting COPD is low and the average age is <60 years in our cohort. COPD impairs lung function and respiratory reserve, leading to difficulties in recovery and a higher risk of post-operative complications^22^.

Previous studies have demonstrated that smokers were more likely to develop PCs and smoking cessation before surgery could help to prevent post-operative complications^10^. However, we did not observe a significant difference in PCs or PPCs between active smokers and quitters. Our observations seemed to indicate that the incremental benefits of smoking cessation might not be prominent among patients at lower risk of developing PCs or PPCs. VATS, mainly when applied to early-stage lung cancer, has been associated with a favorable safety profile with a very low rate of post-operative adverse events^20^. Despite the lack of solid risk stratification of PPCs in thoracic surgery, various important clinical indicators such as age >75 years, the body mass index being ≥30 kg/ m^2^, prolonged surgical time, American Society of Anesthesiology score of 3 or higher, and coexisting COPD, have been shown to correlate with the risks of PPCs^14,23,24^. Although lung function impairment has been identified as the risk factor for PCs in some studies^14,20^, the lack of difference in the incidence of PCs or PPCs when stratified by the magnitude of lung function impairment (e.g. FEV1 pred%) might be attributed to the recruitment of the low-risk population.

Smoking cessation as a potentially modifiable risk factor for PCs has been routinely recommended before surgery. The duration of smoking abstinence before surgery has not been well established although at least four to eight weeks would be preferable^12^. A meta-analysis concluded that longer periods of smoking cessation decreased the incidence of PCs^12^. However, another systematic review has demonstrated no difference in the total incidence of PPCs between current smokers and recent quitters (abstinence for <8 weeks)^25^. In our study, the lack of significant differences in the incidence of PCs, or PPCs in patients receiving elective lung surgery, indicated that advising smokers to quit at any time prior to surgery would not dramatically affect the adverse outcomes for a low-risk population.

Combining pharmacotherapy with counselling or behavioral changes would facilitate smokers to quit smoking^26^. A previous national survey revealed that 73.5% of smokers were aware of the major categories of smoking cessation medications but few had ever used them, which was consistent with the findings of our study^4^. E-cigarettes were the most familiar non-NRT method among patients, but the awareness of effectiveness remained low. The existing clinical trial evidence suggests that e-cigarettes would be effective as the smoking cessation methods, but the safety and efficacy of smoking cessation need to be validated^27^. The American Thoracic Society has endorsed the use of pharmacotherapy with proven efficacy rather than the e-cigarettes for promoting smoking cessation, and the US Preventive Services Task Force has concluded that the evidence is insufficient to fully evaluate the benefits and harms of e-cigarettes for smoking cessation^28,29^. Nicotine dependence may dampen the outcomes of smoking abstinence. For patients with tobacco dependence, e-cigarettes are ineffective and not recommended because of the concerns of maintaining nicotine dependence while having adverse effects^30^. Medications including NRT, bupropion and varenicline have proven efficacy in smoking cessation^31^. In our study, the most well-known NRT methods among patients were nicotine patches and nicotine gum, but the awareness of these methods was <10%. Few patients deemed smoking cessation methods effective, possibly because of various factors, including addiction to nicotine, psychological dependence, lack of access to comprehensive cessation support and inadequate awareness of the benefits of quitting. Addressing these factors through tailored cessation programs, increased access to support services, and enhanced patient education, can improve cessation rates^32^.

The participation of healthcare professionals would augment the outcomes of smoking cessation. Currently, only less than half of Chinese smokers have ever received any advice from physicians for smoking cessation^4^. To mitigate the risk of post-operative complications, 86.6% of patients in the CHAMPION study had received advice from physicians to quit smoking. The relapse rate at six months in this study was <10%, with a greater incidence among quitters. The primary reasons reported for relapse were: ‘I want to smoke when I'm stressed or upse’, and ‘I tried to quit smoking, but it did not work’. These findings remind clinicians to implement more intensified cessation interventions, such as combined pharmacotherapy or behavioral changes. Previous studies showed that 60–75% of smokers experienced relapse within six months^33,34^. In the cases of lung cancer patients who were smokers before surgery, nearly half of them resumed smoking after surgery^35^. Factors such as education level, income, and quit duration before surgery may also influence the likelihood of relapse^35^. Although our cohort demonstrated a relatively low relapse rate compared to previous studies, it is important to note that this observation was based on a short timeframe and a comprehensive evaluation of various impacting factors was not involved.

### Strengths and limitations

The CHAMPION study has provided a broad perspective on smoking cessation patterns based on the data collected from medical charts and the patient- and HCP-reported information. However, there are some limitations that should be considered. First, apart from the limited sample size, some data were missing regarding the cancer stages and lung function metrics. Second, most patients were diagnosed as having lung cancer and our results might not be generalizable to the populations with other pulmonary diseases. Third, recall bias cannot be precluded due to the self-reported smoking assessment questionnaire. Fourth, the generalizability of the study’s results to female patients may be limited given that the findings primarily rely on the experiences and outcomes of male participants. Fifth, the results are based on unadjusted comparisons, potentially introducing bias by not accounting for potential confounding variables. Sixth, continuous abstinence was determined solely through self-report and was not confirmed by objective measures such as CO-oximetry or the determination of cotinine levels in body fluids.

## CONCLUSIONS

Based on our observation, smoking does not markedly increase the risk of post-operative complications among patients undergoing lung surgery and more limited lung resection which may result in a low risk of developing post-operative complications, suggesting that there is likely no need to postpone surgery for those who have not yet quit smoking. However, large-scale randomized controlled studies are necessary to further confirm our findings.

## Data Availability

The datasets used and/or analyzed during the current study are available from the corresponding author on reasonable request.
