# Supplementary material for: Smoking cessation in Chinese patients undergoing thoracic surgery: A multicenter prospective observational study
Source: Tob Induc Dis. 2024 Jan 10;22:10.18332/tid/175639. doi: 10.18332/tid/175639 (PMC10777477; doi:10.18332/tid/175639)
Supplement: Supplementary file 1 [file TID-22-06-s1.pdf]

Supplementary materials

Supplementary Table 1. List of PPCs and other PCs collected

PPCs	Other PCs
Nosocomial pneumonia	Postoperative myocardial infarction
Lobar or whole-lung atelectasis	Low cardiac output syndrome
Acute respiratory failure requiring oxygenation and or intubation	New onset atrial fibrillation
Prolonged air leak	Cardiac arrest or ventricular fibrillation
Pulmonary embolism	Acute kidney injury
Acute Respiratory Distress Syndrome (ARDS)	Stroke
Pneumothorax	Gastrointestinal complications
Bronchospasm requiring use of nebulizer therapy	Incision site infection
Aspiration pneumonitis	Multiple system organ failure
Acute pulmonary edema	Cerebral infarction
Bronchopleural fistula	Cerebral hemorrhage
Bronchitis	Hepatic injury
Pleural effusion	Postoperative hemorrhage
Purulent pleuritis	Unstable angina
Respiratory infection	Sepsis
Chylothorax	Shock
Empyema	Deliriumb
Cough	Stress ulcer
Other, please specify[a]	Circulatory failure
	Reoperation for bleeding
	Electrolyte disturbance
	Fever
	Internal jugular vein thrombosis
	Other, please specify[a]

PC: Postoperative complications, PPC: Postoperative pulmonary complications.
[a]Other PCs or PPCs were determined by the treating physician and the Sponsor.

Supplementary Table 2. List of PCs and PPCs reported

Parameter, N (%)	Total (N = 276)	Smokers (N = 213)	Quitters (N = 63)
**Patients with PCs**	38 (13.8%)	30 (14.1%)	8 (12.7%)
**PCs, occurrence**	48 (17.4%)	38 (17.8%)	10 (15.9%)
Patients with Major PCs	10 (3.6%)	7 (3.3%)	3 (4.8%)
**Major PCs, occurrence**	11 (4.0%)	8 (3.8%)	3 (4.8%)
Pleural effusion	2 (0.7%)	1 (0.5%)	1 (1.6%)
Pneumothorax	2 (0.7%)	1 (0.5%)	1 (1.6%)
Post procedural hemorrhage	2 (0.7%)	2 (0.9%)	0
Cerebral infarction	2 (0.7%)	2 (0.9%)	0
Atelectasis	1 (0.4%)	1 (0.5%)	0
Hyperkalemia	1 (0.4%)	0	1 (1.6%)
Post procedural myocardial infarction	1 (0.4%)	1 (0.5%)	0
Patients with Minor PCs	29 (10.5%)	23 (10.8%)	6 (9.5%)
**Minor PCs, occurrence**	37 (13.4%)	30 (14.1%)	7 (11.1%)
Cough	8 (2.9%)	6 (2.8%)	2 (3.2%)
Pneumonia	6 (2.2%)	5 (2.3%)	1 (1.6%)
Pyrexia	6 (2.2%)	5 (2.3%)	1 (1.6%)
Hypoalbuminemia	3 (1.1%)	2 (0.9%)	1 (1.6%)
Pleural effusion	2 (0.7%)	1 (0.5%)	1 (1.6%)
Asthma	1 (0.4%)	1 (0.5%)	0
Blood lactic acid increased	1 (0.4%)	1 (0.5%)	0
Chylothorax	1 (0.4%)	1 (0.5%)	0
Hypokalemia	1 (0.4%)	1 (0.5%)	0
Hypoproteinemia	1 (0.4%)	1 (0.5%)	0
Metabolic acidosis	1 (0.4%)	1 (0.5%)	0
Noncardiac chest pain	1 (0.4%)	1 (0.5%)	0
Pneumothorax	1 (0.4%)	1 (0.5%)	0
Pulmonary function test decreased	1 (0.4%)	1 (0.5%)	0
Respiratory alkalosis	1 (0.4%)	1 (0.5%)	0
Respiratory tract infection	1 (0.4%)	1 (0.5%)	0
Sputum discolored	1 (0.4%)	0	1 (1.6%)
**Patients with PPCs**	27 (9.8%)	20 (9.4%)	7 (11.1%)
**PPCs, occurrence**	29 (10.5%)	22 (10.3%)	7 (11.1%)
Patients with Major PPCs	6 (2.2%)	4 (1.9%)	2 (3.2%)
**Major PPCs, occurrence**	7 (2.5%)	5 (2.3%)	2 (3.2%)
Pleural effusion	2 (0.7%)	1 (0.5%)	1 (1.6%)
Pneumothorax	2 (0.7%)	1 (0.5%)	1 (1.6%)
Post procedural hemorrhage	2 (0.7%)	2 (0.9%)	0
Atelectasis	1 (0.4%)	1 (0.5%)	0
Patients with Minor PPCs	21 (7.6%)	16 (7.5%)	5 (7.9%)
**Minor PPCs, occurrence**	22 (8.0%)	17 (8.0%)	5 (7.9%)
Cough	8 (2.9%)	6 (2.8%)	2 (3.2%)
Pneumonia	6 (2.2%)	5 (2.3%)	1 (1.6%)
Pleural effusion	2 (0.7%)	1 (0.5%)	1 (1.6%)
Asthma	1 (0.4%)	1 (0.5%)	0
Chylothorax	1 (0.4%)	1 (0.5%)	0
Pneumothorax	1 (0.4%)	1 (0.5%)	0

**Parameter, N (%)**	**Total (N = 276)**	**Smokers (N = 213)**	**Quitters (N = 63)**
Pulmonary function test decreased	1 (0.4%)	1 (0.5%)	0
Respiratory tract infection	1 (0.4%)	1 (0.5%)	0
Sputum discolored	1 (0.4%)	0	1 (1.6%)
**Patients with other PCs**	16 (5.8%)	13 (6.1%)	3 (4.8%)
**Other PCs, occurrence**	19 (6.9%)	16 (7.5%)	3 (4.8%)
Patients with Major Other PCs	4 (1.4%)	3 (1.4%)	1 (1.6%)
Major Other PCs, occurrence	4 (1.4%)	3 (1.4%)	1 (1.6%)
Cerebral infarction	2 (0.7%)	2 (0.9%)	0
Hyperkalaemia	1 (0.4%)	0	1 (1.6%)
Post procedural myocardial infarction	1 (0.4%)	1 (0.5%)	0
Patients with Minor Other PCs	12 (4.3%)	10 (4.7%)	2 (3.2%)
Minor Other PCs, occurrence	15 (5.4%)	13 (6.1%)	2 (3.2%)
Blood lactic acid increased	1 (0.4%)	1 (0.5%)	0
Hypoalbuminaemia	3 (1.1%)	2 (0.9%)	1 (1.6%)
Hypokalaemia	1 (0.4%)	1 (0.5%)	0
Hypoproteinaemia	1 (0.4%)	1 (0.5%)	0
Metabolic acidosis	1 (0.4%)	1 (0.5%)	0
Non-cardiac chest pain	1 (0.4%)	1 (0.5%)	0
Pyrexia	6 (2.2%)	5 (2.3%)	1 (1.6%)
Respiratory alkalosis	1 (0.4%)	1 (0.5%)	0

Percentages are based on the number of all patients within respective columns.
PCs: postoperative complications, PPC: postoperative pulmonary complications.

Supplementary Table 3. Summary of PCs and PPCs among all patients at Month 1, Month 3, and Month 6

**Parameter, N (%)**	**Total (N = 276)**	**Smokers (N = 213)**	**Quitters (N = 63)**
**Month 1**			
**N**	275	212	63
**Patients with PCs**	6 (2.2%)	4 (1.9%)	2 (3.2%)
**PCs, occurrence**	6 (2.2%)	4 (1.9%)	2 (3.2%)
Patients with Major PCs	1 (0.4%)	1 (0.5%)	0
Major PCs, occurrence	1 (0.4%)	1 (0.5%)	0
Pneumonia	1 (0.4%)	1 (0.5%)	0
Patients with Minor PCs	5 (1.8%)	3 (1.4%)	2 (3.2%)
Minor PCs, occurrence	5 (1.8%)	3 (1.4%)	2 (3.2%)
Pneumonia	2 (0.7%)	1 (0.5%)	1 (1.6%)
Bronchitis	1 (0.4%)	0	1 (1.6%)
Cough	1 (0.4%)	1 (0.5%)	0
Pleural effusion	1 (0.4%)	1 (0.5%)	0
**Patients with PPCs**	6 (2.2%)	4 (1.9%)	2 (3.2%)
**PPCs, occurrence**	6 (2.2%)	4 (1.9%)	2 (3.2%)
Patients with Major PPCs	1 (0.4%)	1 (0.5%)	0
Major PPCs, occurrence	1 (0.4%)	1 (0.5%)	0
Pneumonia	1 (0.4%)	1 (0.5%)	0
Patients with Minor PPCs	5 (1.8%)	3 (1.4%)	2 (3.2%)
Minor PPCs, occurrence	5 (1.8%)	3 (1.4%)	2 (3.2%)
Pneumonia	2 (0.7%)	1 (0.5%)	1 (1.6%)
Bronchitis	1 (0.4%)	0	1 (1.6%)
Cough	1 (0.4%)	1 (0.5%)	0
Pleural effusion	1 (0.4%)	1 (0.5%)	0
**Month 3**			
**N**	272	211	61
**Patients with PCs**	3 (1.1%)	2 (0.9%)	1 (1.6%)
**PCs , occurrence**	3 (1.1%)	2 (0.9%)	1 (1.6%)
Patients with Major PCs	0	0	0
Major PCs, occurrence	0	0	0
Patients with Minor PCs	3 (1.1%)	2 (0.9%)	1 (1.6%)
Minor PCs, occurrence	3 (1.1%)	2 (0.9%)	1 (1.6%)
Pleural effusion	1 (0.4%)	1 (0.5%)	0
Pneumothorax	1 (0.4%)	0	1 (1.6%)
Vomiting	1 (0.4%)	1 (0.5%)	0
**Patients with PPCs**	2 (0.7%)	1 (0.5%)	1 (1.6%)
**PPCs , occurrence**	2 (0.7%)	1 (0.5%)	1 (1.6%)
Patients with Major PPCs	0	0	0
Major PPCs, occurrence	0	0	0
Patients with Minor PPCs	2 (0.7%)	1 (0.5%)	1 (1.6%)
Minor PPCs, occurrence	2 (0.7%)	1 (0.5%)	1 (1.6%)
Pleural effusion	1 (0.4%)	1 (0.5%)	0
Pneumothorax	1 (0.4%)	0	1 (1.6%)
**Month 6**			
**N**	271	210	61
**Patients with PCs**	0	0	0
**PCs , occurrence**	0	0	0
Patients with Major PCs	0	0	0

**Parameter, N (%)**	**Total (N = 276)**	**Smokers (N = 213)**	**Quitters (N = 63)**
Major PCs, occurrence	0	0	0
Patients with Minor PCs	0	0	0
Minor PCs, occurrence	0	0	0
**Patients with PPCs**	0	0	0
**PPCs , occurrence**	0	0	0
Patients with Major PPCs	0	0	0
Major PPCs, occurrence	0	0	0
Patients with Minor PPCs	0	0	0
Minor PPCs, occurrence	0	0	0

Percentages are based on the number of all patients within respective columns.
PCs: postoperative complications, PPC: postoperative pulmonary complications.

Supplementary Table 4. Summary of postoperative complications and postoperative pulmonary complications by surgery type

Parameter, N (%)	Lobectomy (N = 162)	Segmentectomy or wedge resection (N = 93)
**Perioperative stage**		
**N**	162	93
**Patients with PCs**	27 (16.7%)	8 (8.6%)
**PCs, occurrence**	34 (21.0%)	11 (11.8%)
Patients with Major PCs	6 (3.7%)	2 (2.2%)
Major PCs, occurrence	7 (4.3%)	2 (2.2%)
Patients with Minor PCs	22 (13.6%)	6 (6.5%)
Minor PCs, occurrence	27 (16.7%)	9 (9.7%)
**Patients with PPCs**	20 (12.3%)	5 (5.4%)
**PPCs, occurrence**	21 (13.0%)	6 (6.5%)
Patients with Major PPCs	5 (3.1%)	0
Major PPCs, occurrence	6 (3.7%)	0
Patients with Minor PPCs	15 (9.3%)	5 (5.4%)
Minor PPCs, occurrence	15 (9.3%)	6 (6.5%)
**Month 1**		
**N**	161	93
**Patients with PCs**	3 (1.9%)	3 (3.2%)
**PCs, occurrence**	3 (1.9%)	3 (3.2%)
Patients with Major PCs	1 (0.6%)	0
Major PCs, occurrence	1 (0.6%)	0
Patients with Minor PCs	2 (1.2%)	3 (3.2%)
Minor PCs, occurrence	2 (1.2%)	3 (3.2%)
**Patients with PPCs**	3 (1.9%)	3 (3.2%)
**PPCs, occurrence**	3 (1.9%)	3 (3.2%)
Patients with Major PPCs	1 (0.6%)	0
Major PPCs, occurrence	1 (0.6%)	0
Patients with Minor PPCs	2 (1.2%)	3 (3.2%)
Minor PPCs, occurrence	2 (1.2%)	3 (3.2%)
**Month 3**		
**N**	160	91
**Patients with PCs**	3 (1.9%)	0
**PCs, occurrence**	3 (1.9%)	0
Patients with Major PCs	0	0
Major PCs, occurrence	0	0
Patients with Minor PCs	3 (1.9%)	0
Minor PCs, occurrence	3 (1.9%)	0
**Patients with PPCs**	2 (1.3%)	0
**PPCs, occurrence**	2 (1.3%)	0
Patients with Major PPCs	0	0
Major PPCs, occurrence	0	0
Patients with Minor PPCs	2 (1.3%)	0
Minor PPCs, occurrence	2 (1.3%)	0
**Month 6**		
**N**	160	91
**Patients with PCs**	0	0

**Parameter, N (%)**	**Lobectomy (N = 162)**	**Segmentectomy or wedge resection (N = 93)**
**PCs, occurrence**	0	0
Patients with Major PCs	0	0
Major PCs	0	0
Patients with Minor PCs	0	0
Minor PCs	0	0
**Patients with PPCs**	0	0
**PPCs, occurrence**	0	0
Patients with Major PPCs	0	0
Major PPCs, occurrence	0	0
Patients with Minor PPCs	0	0
Minor PPCs, occurrence	0	0

Percentages are based on the number of all patients within respective columns.
PCs: postoperative complications, PPC: postoperative pulmonary complications.

Supplementary Table 5. Summary of postoperative complications and postoperative pulmonary complications by surgery type

Parameter, N (%)	FEV1% ≥ 80% (N = 62)	FEV1% < 80% (N = 172)
**Peri-operative Stage**		
**N**	62	172
**PCs, occurrence**	15 (24.2%)	28 (16.3%)
**Patients with PCs**	12 (19.4%)	22 (12.8%)
**Major PCs, occurrence**	2 (3.2%)	8 (4.7%)
Patients with Major PCs	2 (3.2%)	7 (4.1%)
Atelectasis	0	1 (0.6%)
Cerebral infarction	0	2 (1.2%)
Hyperkalemia	1 (1.6%)	0
Pleural effusion	0	1 (0.6%)
Pneumothorax	0	2 (1.2%)
Post procedural hemorrhage	0	2 (1.2%)
Post procedural myocardial infarction	1 (1.6%)	0
**Minor PCs, occurrence**	13 (21.0%)	20 (11.6%)
Patients with Minor PCs	10 (16.1%)	15 (8.7%)
Asthma	0	1(0.6%)
Blood lactic acid increased	0	1 (0.6%)
Cough	2 (3.2%)	6 (3.5%)
Hypoalbuminemia	1 (1.6%)	2 (1.2%)
Hypokalemia	1 (1.6%)	0
Hypoproteinemia	1 (1.6%)	0
Metabolic acidosis	0	1 (0.6%)
Non-cardiac chest pain	1 (1.6%)	0
Pleural effusion	0	1 (0.6%)
Pneumonia	4 (6.5%)	2 (1.2%)
Pneumothorax	1 (1.6%)	0
Pulmonary function test decreased	0	1 (0.6%)
Pyrexia	2 (3.2%)	3 (1.7%)
Respiratory alkalosis	0	1 (0.6%)
Respiratory tract infection	0	1 (0.6%)
**PPCs, occurrence**	7 (11.3%)	18 (10.5%)
**Patients with PPCs**	7 (11.3%)	16 (9.3%)
**Major PPCs, occurrence**	0	6 (3.5%)
Patients with Major PPCs	0	5 (2.9%)
Atelectasis	0	1 (0.6%)
Pleural effusion	0	1 (0.6%)
Pneumothorax	0	2 (1.2%)
Post procedural hemorrhage	0	2 (1.2%)
**Minor PPCs, occurrence**	7 (11.3%)	12 (7.0%)
Patients with Minor PPCs	7 (11.3%)	11 (6.4%)
Asthma	0	1 (0.6%)
Cough	2 (3.2%)	6 (3.5%)
Pleural effusion	0	1 (0.6%)
Pneumonia	4 (6.5%)	2 (1.2%)
Pneumothorax	1 (1.6%)	0
Pulmonary function test decreased	0	1 (0.6%)
Respiratory tract infection	0	1 (0.6%)
**Other PCs, occurrence**	8 (12.9%)	10 (5.8%)
**Patients with Other PCs**	7 (11.3%)	8 (4.7%)
**Major Other PCs, occurrence**	2 (3.2%)	2 (1.2%)
Patients with Major Other PCs	2 (3.2%)	2 (1.2%)
Cerebral infarction	0	2 (1.2%)
Hyperkalemia	1 (1.6%)	0
Post procedural myocardial infarction	1 (1.6%)	0
**Minor Other PCs, occurrence**	6 (9.7%)	8 (4.7%)
Patients with Minor Other PCs	5 (8.1%)	6 (3.5%)
Blood lactic acid increased	0	1 (1.6%)
Hypoalbuminemia	1 (1.6%)	2 (1.2%)
Hypokalemia	1 (1.6%)	0
Hypoproteinemia	1 (1.6%)	0

Metabolic acidosis	0	1 (1.6%)
Non-cardiac chest pain	1 (1.6%)	0
Pyrexia	2 (3.2%)	3 (1.7%)
Respiratory alkalosis	0	1 (1.6%)
**Month 1**		
**N**	62	171
**PCs (including other PCs), occurrence**	1 (1.6%)	4 (2.3%)
**Patients with PCs (including other PCs)**	1 (1.6%)	4 (2.3%)
**Major PCs, occurrence**	0	0
Patients with Major PCs	0	0
**Minor PCs, occurrence**	1 1.6%)	4 (2.3%)
Patients with Minor PCs	1 (1.6%)	4 (2.3%)
Bronchitis	0	1 (0.6%)
Cough	0	1 (0.6%)
Pleural effusion	0	1 (0.6%)
Pneumonia	1 (1.6%)	1 (0.6%)
**PPCs (including other PPCs), occurrence**	1 (1.6%)	4 (2.3%)
**Patients with PPCs (including other PPCs)**	1 (1.6%)	4 (2.3%)
**Major PPCs, occurrence**	0	0
Patients with Major PPCs	0	0
**Minor PPCs, occurrence**	1 (1.6%)	4 (2.3%)
Patients with Minor PPCs	1 (1.6%)	4 (2.3%)
Bronchitis	0	1 (0.6%)
Cough	0	1 (0.6%)
Pleural effusion	0	1 (0.6%)
Pneumonia	1 (1.6%)	1 (0.6%)
**PPCs (including other PPCs), occurrence**	1 (1.6%)	4 (2.3%)
**Patients with PPCs (including other PPCs)**	1 (1.6%)	4 (2.3%)
**Major PPCs, occurrence**	0	0
Patients with Major PPCs	0	0
**Minor PPCs, occurrence**	1 (1.6%)	4 (2.3%)
Patients with Minor PPCs	1 (1.6%)	4 (2.3%)
Bronchitis	0	1 (0.6%)
Cough	0	1 (0.6%)
Pleural effusion	0	1 (0.6%)
Pneumonia	1 (1.6%)	1 (0.6%)
**Month 3**		
**N**	62	169
**PCs (including other PCs), occurrence**	0	1 (0.6%)
**Patients with PPCs (including other PPCs)**	0	1 (0.6%)
**Major PPCs, occurrence**	0	0
Patients with Major PPCs	0	0
**Minor PPCs, occurrence**	0	1 (0.6%)
Patients with Minor PPCs	0	1 (0.6%)
Pleural effusion	0	1 (0.6%)
**Month 6**		
**N**	62	168
**PCs (including other PCs), occurrence**	0	0
**Patients with PCs (including other PCs)**	0	0
**Major PCs, occurrence**	0	0
Patients with Major PCs	0	0
**Minor PCs, occurrence**	0	0
Patients with Minor PCs	0	0
**PPCs (including other PPCs), occurrence**	0	0
**Patients with PPCs (including other PPCs)**	0	0
**Major PPCs, occurrence**	0	0
Patients with Major PPCs	0	0
**Minor PPCs, occurrence**	0	0
Patients with Minor PPCs	0	0

Percentages are based on the number of all patients within respective columns.

Peri-operative Stage is defined as 1 day prior to discharge or within 2 weeks after surgery, whichever occurs first. PCs: postoperative complications, PPC: postoperative pulmonary complications.

Supplementary Figure 1. NRT and non-NRT smoking cessation methods heard or tried in the patients and HCPs

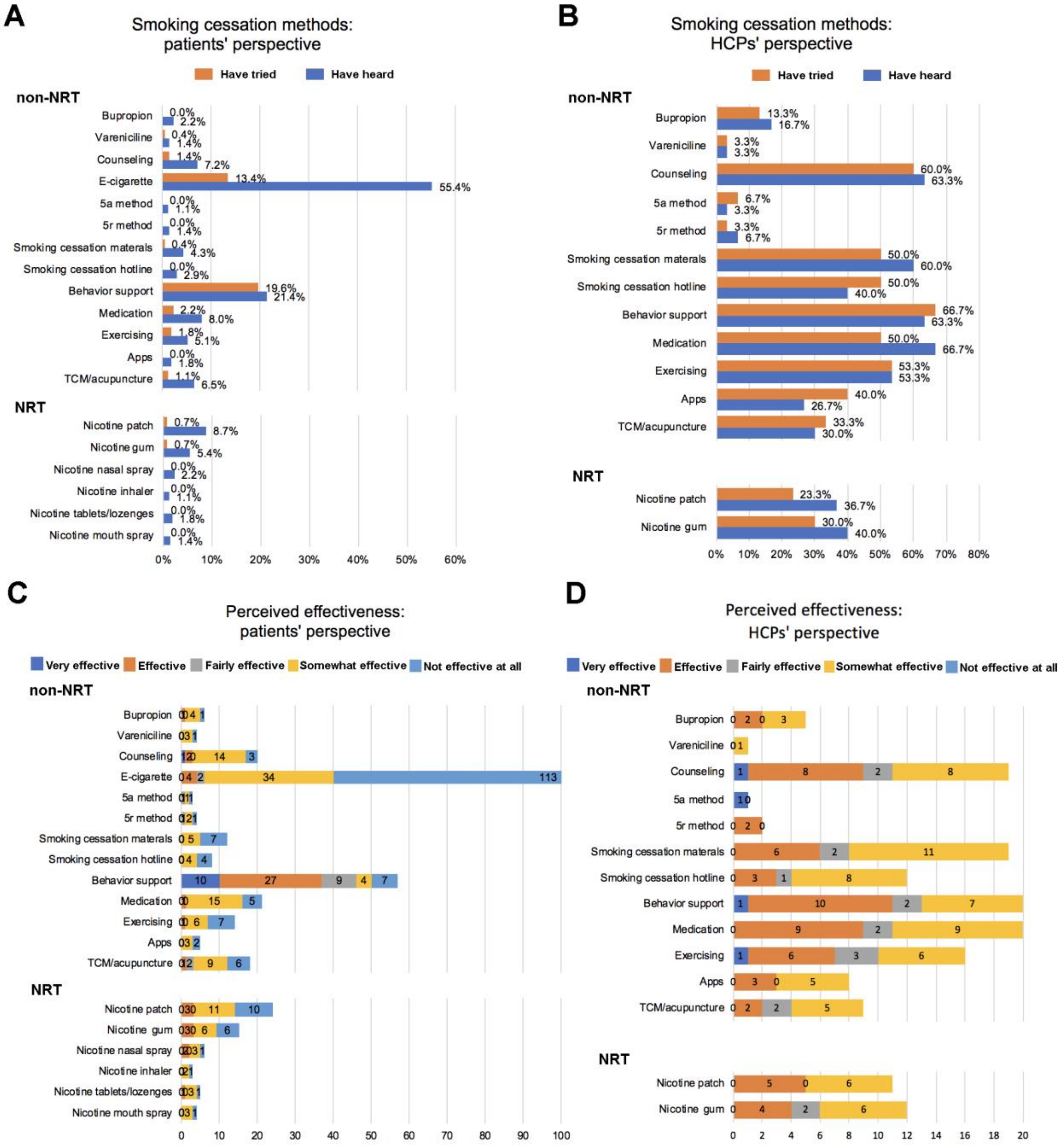

©2024 He J. et al.
